# The analysis of prognostic factors of primary small intestinal gastrointestinal stromal tumors with R0 resection: A single-center retrospective study

**DOI:** 10.1097/MD.0000000000029487

**Published:** 2022-06-24

**Authors:** Hui Qu, ZhaoHui Xu, YanYing Ren, ZeZhong Gong, Ri Hyok Ju, Fan Zhang, Shuai Shao, XiaoLiang Chen, Xin Chen

**Affiliations:** a Department of Hernia and Colorectal Surgery, The Second Hospital of Dalian Medical University, Dalian, People's Republic of China; b Dalian Medical University, Dalian, People's Republic of China.

**Keywords:** gastrointestinal stromal tumors, GIST, imatinib therapy, Ki67, prognosis, small intestine, tumor rupture

## Abstract

**Objective:**

We aim to assess factors that affect overall survival in patients with primary small intestinal gastrointestinal stromal tumors (GISTs) who had undergone R0 resection.

**Method:**

A retrospective analysis reviewed the data of 82 consecutive confirmed GIST patients at a single medical center in China from January 2012 to June 2020. The survival curve was estimated using the Kaplan–Meier method, and independent prognostic factors were confirmed using the Cox regression model.

**Results:**

A total of 82 patients were included in the study: 42 men and 40 women, the mean age was 59 years old (23–83 years old). Tumors were commonly found in the jejunum (46.3%), ileum (20.7%), and duodenum (32.9%). The median tumor size was 6.0 cm (range: 1.0–15.0 cm). The number of mitoses per one 50 high-power field was used to define the mitotic rates. In our present study, 56 patients presented a mitotic rate ≤5 (68.3%) and 26 patients showed a rate >5 (31.7%) at the time of diagnosis. All patients accepted tumor resection without lymph node resection. The positivity rate was 97.6% for CD117, 96.3% for delay of germination 1, 65.9% for CD34, 6.1% for S-100, and 59.8% for smooth muscle actin using immunohistochemistry. Tumor size, tumor rupture, Ki67 index, mitotic index, and postoperative imatinib were independent prognostic factors for small intestinal GISTs.

**Conclusions:**

In this study, larger tumor size, high Ki67 index, high mitotic index, the occurrence of tumor rupture, and use of imatinib were independent unfavorable prognostic indicators.

## 1 Introduction

Gastrointestinal stromal tumor (GIST) is the most common mesenchymal tumor of the gastrointestinal tract, with an annual incidence of 10 to 15 cases per million.^[1,2]^ GISTs are believed to originate from the pacemaker cells in the intestinal tract called Cajal interstitial cells. Activating mutations in KIT or PDGFRA oncogenes are considered the key molecular drivers of GIST pathogenesis.^[3]^ Most GISTs originate in the stomach (50%–60%), followed by the small intestine (20%–30%), colon or rectum (5%–10%), the esophagus <(5%), and occasionally outside the gastrointestinal tract.^[4,5]^ In recent years, imatinib, a selective protein tyrosine kinase inhibitor, has been developed as a targeted molecular therapy for GISTs. To date, imatinib is the first-line standard therapy for metastatic or recurrent GISTs.^[6,7]^ Complete surgical resection is still the best treatment modality as approximately 60% of patients with localized primary GIST are cured with resection (R0).^[8]^ However, DeMatteo et al reported that, in more than half of these patients, the disease reappeared within 5 years of surgery.^[9]^

Several risk-stratification studies have established independent risk factors for operable GISTs. The modified NIH (National Institutes of Health) classification proposed by Joensuu and colleagues of is the most accepted classification in clinical practice, as it combines the advantages of the NIH and Armed Forces Institute of Pathology (AFIP) criteria with the rupture before or during surgery.^[10]^ However, an accurate risk prediction in patients with GIST who underwent surgery using the modified NIH classification alone may be insufficient. Moreover, several studies found that Ki67 expression is associated with the prognosis of postoperative GISTs.^[11–13]^ Guidelines of the European Society for Medical Oncology (ESMO; The ESMO/European Sarcoma Network Working Group, 2018) and the National Comprehensive Cancer Network (NCCN) recommend that imatinib is the standard treatment for patients with a significant risk of relapse by adjuvant imatinib therapy for 3 years. When the risk is intermediate, a shared decision-making process is needed.^[14]^ The relationship of imatinib therapy with clinical benefit in GIST patients remain poorly defined.

The small intestine is the second most common site of the appearance of GIST. Studies have shown that small intestinal GISTs have a worse prognosis than primary gastric GISTs.^[15,16]^ The diagnosis of small intestine GISTs is easily delayed for several reasons, such as insidious onset, nonspecific clinical manifestations, and challenging examination. Hence, in the imatinib era, identifying of independent prognostic indicators is critical for accurately assessing risk stratification, which may help determine the strategies of imatinib adjuvant therapy and postoperative follow-up. In our research, to better understand the prognostic factors and improve the risk stratification assessment, we analyzed the immunohistochemical expression and clinicopathological characteristics of a series of small intestinal GISTs with complete resection at our center.

## 2 Materials and methods

### 2.1 Study population

**Figure 1:**
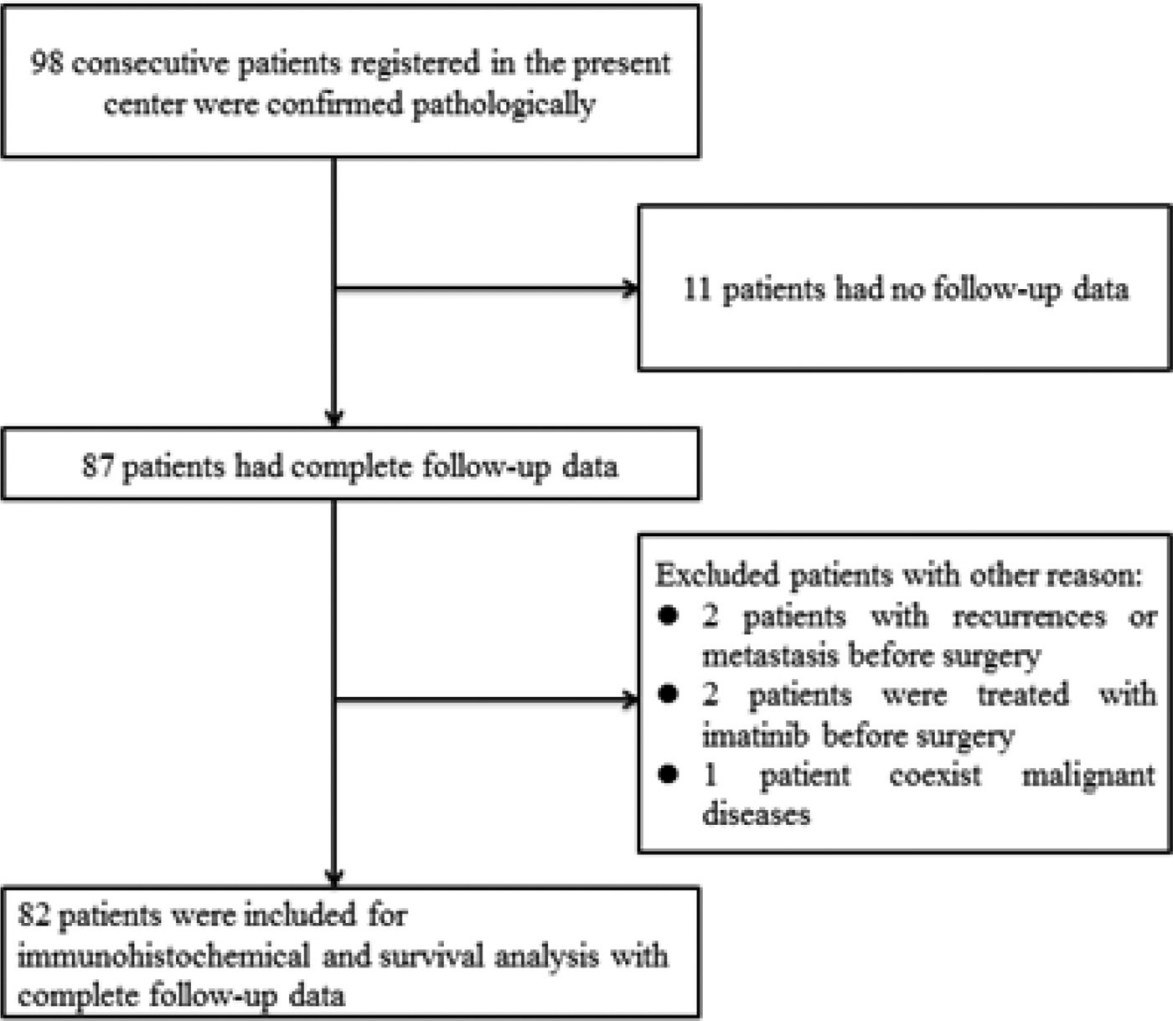
Flow chart of patient selection.

All patients with small intestinal GISTs registered in the general surgery department of the Second Hospital of Dalian Medical University, Dalian, China, from January 2012 to June 2020 were collected for examination. All patients who underwent resection had negative surgical margins. The inclusion criteria of this study are as follows: (1) no recurrences or metastasis before surgery; (2) no coexisting malignant diseases; (3) patients underwent open R0 resection or laparoscopic surgery; (4) patients were not treated with imatinib, chemotherapy or radiotherapy before surgery; (5) access to complete follow-up data were complete. The process of patient selection is shown in Figure 1.

### 2.2 Follow-up

The regular follow-up data of all 82 patients were retrieved from course/patients records. Follow-up data were collected once a year for patients with less than the intermediate risk of recurrence or, twice a year for medium or high-risk patients. Endoscopy, abdominopelvic computed tomography scan, and abdominal ultrasound were performed on every patient. The clinical examination and pathological results were used to diagnose recurrence or metastasis.

Follow-up was performed by outpatient review and telephone calls, and the last follow-up was completed in June, 2020. Overall survival (OS) was defined as the time from the date of R0 resection to the date of any death or the last available follow-up.

### 2.3 Ethics statement

This study, ’prognostic analysis factors of primary small intestinal gastrointestinal stromal tumors with R0 resection’ was approved by the Ethics Committees of the Second Affiliated Hospital of the Dalian Medical University (Dalian, China). As a retrospective study, informed consent is not required from participants. The Ethical Committee found that informed consent is not necessary and that all samples and information were incognito.

### 2.4 Statistical analysis

All statistical calculations were done with the IBM SPSS statistical software package (Version 25.0; IBM Corp, New York, NY). Descriptive data were presented as mean, and classification data were expressed with a 95% confidence interval. Survival analyzes were performed using Kaplan–Meier analysis, and survival distributions were tested using a log-rank test among every group. The case of *P*-value <.100 in the Kaplan–Meier analysis was incorporated in the forward stepwise Cox proportional hazards regression model. Predictive factors were determined depending on cox regression analysis. A two-sided *P*-value <.05 was considered statistically significant.

## 3 Results

### 3.1 Tumor features and Immunohistochemical expression

All 82 patients with GISTs were analyzed in the study as shown in Table 1. The total cohort included 42 (51.2%) men and 40 (48.8%) women. The age range was 23 to 83 years (median: 59 years). There were 43 patients (52.4%) who were older than 60 years. Tumor sites included the jejunum (46.3%), ileum (20.7%), and duodenum (33.0%). The median tumor size was 6.0 cm (range: 1.5–15.0 cm), 38 cases (46.3%) were ≤5 cm, 32 cases (39.0%) were 5.1 to 10 cm and 12 cases (14.6%) were >10 cm. The mitotic count of 56 patients was ≤5 (68.3%) and 26 patients were >5 (31.7%). Moreover, 10 of 82 patients (12.2%) had tumor rupture. The positivity rate of immunohistochemistry was 97.6% for CD117, 96.3% for delay of germination 1 (DOG-1), 65.9% for CD34, 6.1% for S-100, and 59.8% for smooth muscle actin. The Ki67 index of 49 cases was ≤5%, and 33 cases were > 5%. The histological subtypes included spindle (n = 67; 81.7%), epithelioid (n = 9; 11.0%) and mixed (n = 6; 7.3%) types. According to the modified NIH classification, 4 (4.9%) cases of very low, 26 (31.7%) low, 4 (4.9%) intermediate and 48 (58.5%) high-risk tumors.

**Table 1:** Patients’ characteristics and univariate analysis.

	Patients’ demographic and clinical characteristics	Univariate analysis of OS
Variables	n	%	χ^2^	*P*
Gender			0.097	.756
Male	42	51.2		
Female	40	48.8		
Age (y)			3.394	.065
≤60	43	52.4		
>60	39	47.6		
Tumor size (cm)			7.111	.029
≤5	38	46.4		
5.1–10	32	39.0		
>10	12	14.6		
Mitotic index (/50 HPF)			9.001	.003
≤5	56	68.3		
>5	26	31.7		
Operation procedure			0.282	.595
Open	59	72.0		
Laparoscopy	23	28.0		
Clinical symptom			3.586	.465
Abdominal pain	40	48.8		
Bleeding	24	29.3		
Abdominal mass	12	14.6		
Asymptomatic	4	4.9		
Others	2	2.4		
NIH risk score			7.031	.071
Very low	4	4.9		
Low	25	31.7		
Moderate	4	4.9		
High	42	58.5		
Histological type			4.274	.118
Spindle	67	81.7		
Epithelioid	9	11.0		
Mixed	6	7.3		
Imatinib therapy			0.142	.706
Absent	56	68.3		
Present	26	31.7		
CD117			0.289	.591
Positive	80	97.6		
Negative	2	2.4		
DOG-1			0.207	.649
Positive	79	96.3		
Negative	3	3.7		
CD34			0.113	.737
Positive	54	65.9		
Negative	28	34.1		
SMA			2.224	.136
Positive	50	61.0		
Negative	32	39.0		
S-100			0.069	.792
Positive	77	93.9		
Negative	5	6.1		
Ki67			9.768	.002
≤5%	49	59.8		
>5%	33	40.2		
Tumor rupture			16.824	<.001
Absent	72	87.8		
Present	10	12.2		
Tumor location			1.291	.525
Duodenum	27	32.9		
Jejunum	38	46.3		
Ileum		17	20.7	

DOG-1 = delay of germination 1, HPF = high power field, NIH = National Institutes of Health, OS = overall survival, SMA = smooth muscle actin.

### 3.2 Survival analysis

**Figure 2:**
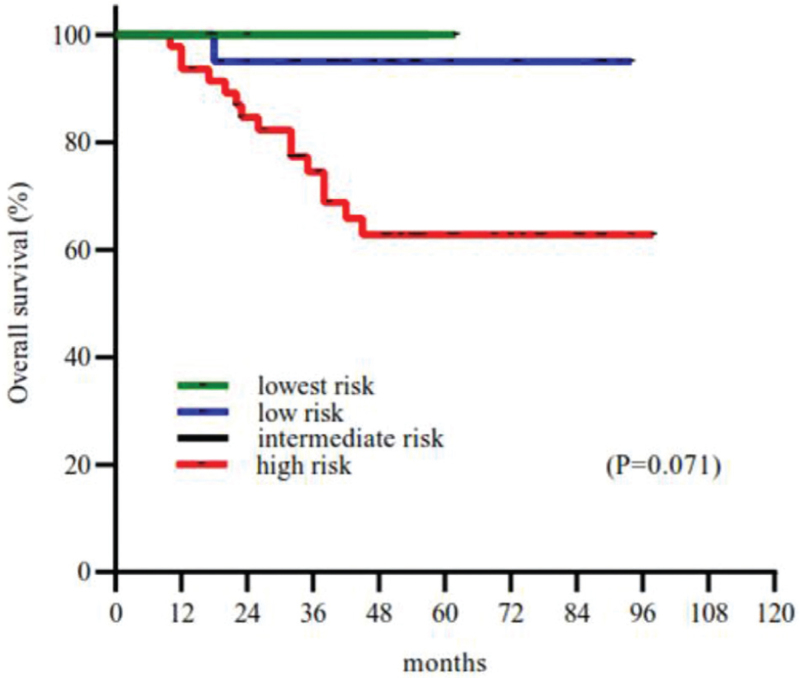
Overall survival curves based on the modified National Institutes of Health Classification.

**Figure 3:**
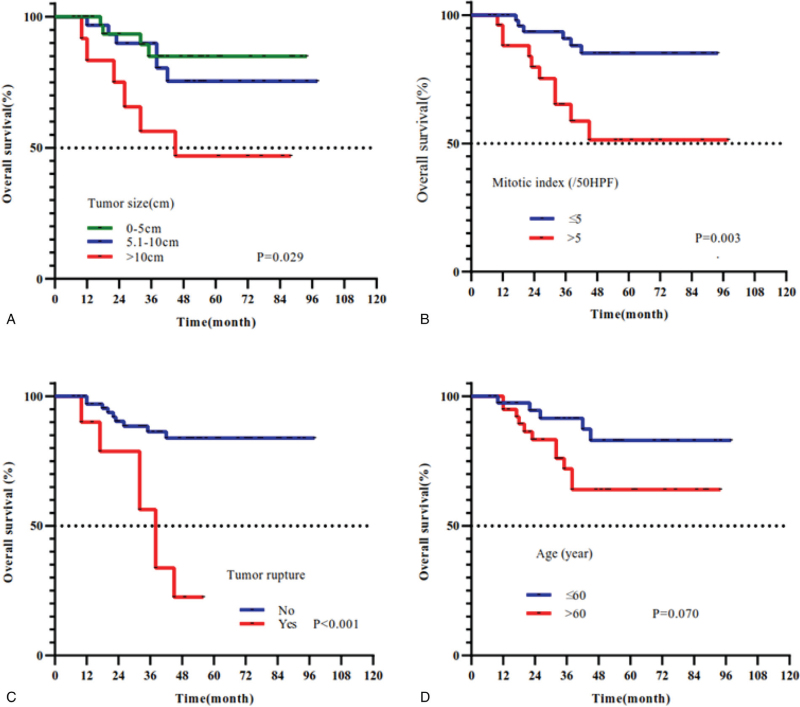
Overall survival curves based on postoperative imatinib for all patient administration in (A) (*P* = .701); for intermediate and high risk patients in (B) (*P* = .094).

**Figure 4:**
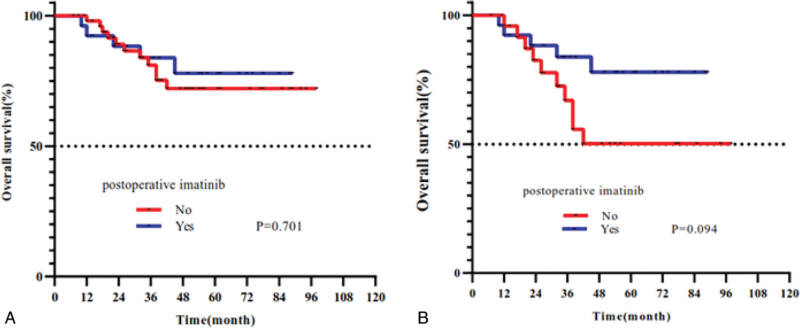
Overall survival curves for patients with primary small intestinal gastrointestinal stromal tumors based on (A) tumor size (*P* < .029); (B) mitotic index (*P* = .003); (C) tumor rupture (*P* < .001), and (D) age (*P* = .070).

Complete follow-up data were obtained for all 82 patients who underwent R0 resection. The median time of follow-up duration was 43 months (range: 7–98). According to the modified NIH classification, there were 4 cases of very low (4.9%), 26 low (31.7%), 4 intermediate (4.9%), and 48 high (58.5%) risk tumors. The 5 year survival rate in very low-risk, low, intermediate, and high-risk patients was 100%, 96.2%, 100%, and 68.8%, respectively, by the modified NIH classification (Fig. 2). The analysis index such as tumor size, Ki67 index, tumor rupture, mitotic index, and age were all related to OS by univariate analysis (all *P* ≤ .1). However, sex, clinical symptom, postoperative imatinib therapy, and other parameters showed no significant statistical differences for the prognosis (All *P* > .1) (Table 1). Furthermore, two intermediate-risk and 22 high-risk patients received oral adjuvant imatinib (400 mg/d). Considering the guidelines that suggested that adjuvant imatinib therapy was used in the postoperative intermediate-risk patients for 1 year at least and high-risk patients for 3 years until unacceptable side effects occurred or disease were progressed, we further used the Kaplan–Meier analyses method in these patients with OS (Fig. 3). The Kaplan–Meier curve showed that surgery together with postoperative imatinib resulted in better survival than only surgery (OS: 80.8% vs. 61.5%; *P* < .1) in intermediate and high-risk patients. Therefore, we included imatinib therapy in the multivariate analysis. Our analysis showed that surgery plus postoperative imatinib was an independent predictor [hazard ratio: 0.180; *P* = .027]. Furthermore, larger tumor size, Ki67 index, tumor rupture, and mitotic index were also identified as independent prognostic factors of OS (all *P* < .05; Fig. 4, Table 2).

**Table 2:** Multivariate analysis for overall survival.

Variables	DF	HR	95.0% (HR) CI	*P*
Age	1	2.243	0.693–7.259	.178
Tumor size (cm)
0–5	2			
5.1–10	1	1.351	0.373–4.902	.647
>10	1	5.374	1.103–26.175	.037
Mitotic index (>5/HPF)	1	3.655	1.033–12.926	.044
Ki67 index	1	4.308	1.282–14.473	.018
Imatinib therapy	1	0.180	0.040–0.819	.027
tumor rupture	1	2.901	1.033–8.150	.043

95% CI = 95% confidence interval, DF = degree of freedom, HPF = high power field, HR = hazard ratio.

## 4 Discussion

Although GISTs are a relatively rare disease, a large number of trials and consensus conferences have improved the strategies of patients with GISTs for diagnosis, treatment, and follow-up. However, these trials were performed on GIST of all location and some subgroup analysis was reported at different locations. Although the small intestine is the second most common site for the development of GISTs, data to evaluate the prognosis in patients undergoing resection of R0 GIST originating from the small intestine are relatively inadequate. Due to their heterogeneity, small intestinal GISTs require individualized diagnosis and treatment.^[17]^ Imatinib, the first-line drug for advanced GIST-targeted therapy, dramatically improves patient survival. The NCCN^[18]^ recommends imatinib as adjuvant therapy for at least 36 months after GIST resection for patients at high risk of recurrence. Given the use of adjuvant therapy, it is a significant challenge to determine which patients are the most likely to experience metastasis or recurrence. Therefore, our study retrospectively collected clinicopathological and immunohistochemical characteristics of 82 patients with small intestinal GISTs who underwent R0 resection at our center for further analysis. These results might help the strategies for more personalized therapeutic and follow-up.

As with all retrospective studies, missing data were unavoidable in the progress of follow-up. As such, there are inherent limitations that cannot be entirely overcome including omitted variables and selection bias. Among the patients we followed up, 16 were lost to follow-up for different reasons, which may lead to selection bias, and loss of follow-up may lead to a small number of samples, which may have a particular impact on the accuracy of the study results. We included as many cases as possible and use all available information to reduce bias. In the present study, the basic clinic pathologic features of small intestinal GISTs are consistent with previous studies including similar immunohistochemical expression and tumor characteristics.^[19–21]^ Joensuu et al reported parallel demographic data of GISTs. They found that the median age of patients with GIST was 64 years with a slight advantage for men.^[22]^ Another recent study, the first attempt to determine and estimate the global burden of GISTs, also found that the sex distribution of GIST patients shows a considerably coincident equal distribution between males and females with the median age 60s.^[2]^ In our patients, we found a similar distribution between males and females. Our study also showed a similar median age to Western studies with a median age of 59 years. In fact, several studies also showed that age was an independent predictor of affecting prognosis significantly.^[23,24]^ However, in the present study, both age and sex were not significantly associated with OS (*P* = .065; *P* = .756).

The morphology of GIST is variable, and therefore immunohistochemical staining of markers is essential for the accurate diagnosis and differential diagnosis of GIST. Additionally, the different antibodies were summarized in a recent study that showed that CD117 (90%–95%), DOG1 (98%), and CD34 (80%–85%) for gastric or (50%) for small intestinal GIST are the most useful.^[25]^ Our results are identical with these studies through the expression with CD117 (97.6%), DOG1 (96.3%), and CD34 (65.9%) in the present study.

Several studies suggested that immunohistochemical expression is related to the prognosis. Rizzo et al suggested that the strong expression of DOG1 is relevant to poor disease-free survival, revealing its potential ability to predict the detrimental prognosis of GISTs.^[26]^ Miettinen et al also reported that smooth muscle actin positivity was a favorable factor.^[27]^ Simultaneously, a few other studies reported that a high Ki67 positive index might be a potential negative prognostic predictor for outcome in GISTs.^[11,13,28]^ Nevertheless, except for the Ki67 index, we found no association of other immunohistochemical expressions with OS in our patients. Ki67, a human nuclear cell proliferation-associated antigen is expressed in the synthesis and growth phases of the entire cell cycle, not during the resting phase.^[29]^ On the contrary, the mitotic index only expressed the M phase. It may be a potential indicator that affects the prognosis of GISTs after the mitotic index. However, a consistent methodology is needed for testing in further studies. Moreover, the cut-off point for Ki67 positivity is varied. The recent studies, Jeong et al,^[30]^ and Seven et al,^[12]^ furtherly showed the association between disease-free survival and Ki67 positivity and thought the optimal cut-off value of the Ki67 index was 5%.

In our study, we used the same cut-off point (5%), and univariate analysis showed that the Ki67 index (>5%) was an independent prognostic predictor. The multivariate analysis found that it may have a potential prognostic value (*P* = .018). A high Ki67 positive index is associated with worse OS. Although immunohistochemical staining markers play a crucial role in diagnosing GISTs, the relationship between immunohistochemical expression and OS remains uncertain.

Independent risk factors have been extensively over the 20 years. Several risk-stratification criteria have been widely accepted and applied in clinical practice. The first system, the consensus criteria of the NIH, was published in 2002, with the two most crucial prognostic factors, including the mitotic index and tumor size.^[31]^ GIST location was added as a third risk factor following the tumor size and mitotic count by the AFIP criteria. After Rutkowski et al first documented tumor rupture as an independent risk factor for recurrence,^[32]^ the modified NIH classification included the superiority of the NIH and AFIP criteria along with the factor of tumor rupture before or during surgery. The two most clarified prognostic factors, size and mitotic rate, were confirmed by multicenter studies. Although the mitotic rate of 5 per 50 high-power field (HPF) is widely used as a cut-off value to risk stratify GISTs,^[33]^ a recent study, the first SEER trend analysis, assessed 5138 patients with GIST, although a cut-off value of 5 cm size was used to predict a worse prognosis may be over-pessimism.

Although the mitotic rate of 5 per 50 (HPF) is used as a cut-off value to the risk GISTs widely,^[33]^ a recent study, the first SEER trend analysis, assessed 5138 patients with GIST, thought that a 5 cm size cut-off value was used to predict a worse prognosis.

They suggested that a tumor size greater than 10 cm was related to worse OS in the multivariate analysis.^[34]^ Moreover, another study also suggested that size above 10 cm was also related to a worse prognosis.^[35]^ In our study, similar results of the multivariate analysis also showed that size above 10 cm and a mitotic rate of 5 per 50 HPF was also related to worse OS in small intestinal GISTs patients (*P* = .037; *P* = .044).

However, tumor rupture is the most widely investigated among these prognostic factors. In a recent study, Nishida et al collected 665 patients with primary GISTs who had undergone R0 or R1 resection. In this study, tumor rupture was an independent prognostic factor for RFS, but not for OS, in the time of imatinib.^[36]^ Several studies found that tumor rupture is more frequent in small intestinal than in gastric GIST.^[37,38]^ In a European study, Rutkowski, et al found that GIST with tumor rupture usually had more adverse prognostic features, such as larger volume, high mitotic count, and non-gastric source sites.^[38]^ However, there is no consistent or generally accepted definition for the term ’tumor rupture’, and its incidence has varied widely among reported series. Based on the Oslo criteria, Nishida and colleagues came up with six definitions for rupture.^[39]^ Ten patients had tumor rupture in our study. We also follow definitions of tumor rupture to investigate the relationship between rupture and OS. Multivariate analysis showed the occurrence of tumor rupture in GIST of small intestine had an independent negative influence on prognosis. Although the modified NIH risk assessment may probably be considered as the standard for pathologists and clinicians, it may still help to ameliorate the administration of the small intestinal GISTs by continuing to seek other prognostic factors.

The two guidelines of the NCCN and ESMO suggested the employ of adjuvant imatinib therapy for GISTs patients with middle-risk for 1 year and high-risk for 3 years. Guidelines also pointed out that the data available is insufficient to direct the use of adjuvant imatinib in these studies. Several studies showed that the survival time of 3 years of imatinib therapy is better than that of 1 year of imatinib therapy.^[40,41]^ Another recent study that included 1559 patients showed that adjuvant treatment is connected with a significant survival preponderance in patients presenting with high-grade GISTs that are >5 cm in size and >5 mitoses/50 HPF.^[17]^ Liu and co-workers found that adjuvant imatinib improved high-risk patients significantly, but intermediate-risk patients did not.^[42]^ In our study, univariate analysis showed the imatinib is not significant. Given that the guidelines suggested adjuvant treatment for surgical patients with intermediate and high-risk recurrence, we also applied a Kaplan–Meier analysis in intermediate-risk and high-risk patients with OS. We found that surgery combining postoperative imatinib adjuvant therapy got a better survival outcome than only surgery (*P* < .1). Therefore, we included the variables (imatinib therapy) in our multivariate analysis and showed that imatinib therapy was a positive independent predictor of prognosis (*P* = .027). Therefore, we suggested that the use of imatinib adjuvant for the patients who had undergone surgery. However, adjuvant therapy for GISTs, particularly in small intestinal GISTs, prospective multicenter randomized controlled trials to further formulate adjuvant therapy strategies will guide our clinical work and enable us to achieve standardized, individualized diagnosis strategies, treatment, and follow-up for small intestinal GISTs.

There are some limitations to our study. Our patients were retrospectively collected from a single center and the number of patients was less than in some published studies. As such, there are inherent limitations that cannot be entirely overcome. Our follow-up period is from 2012 to 2020, which has a long time span. Our study also lacked the effect of mutation type on the prognosis. Therefore, a well-designed multi-center cohort study with a larger sample size was worthy of further investigation. Furthermore, some recent studies showed that mutant types of GIST could provide potential prognostic information for risk assessment.^[43,44]^ Regrettably, data were insufficient to investigate the relationship between mutant types and prognosis.

## 5 Conclusions

In the present study, tumor size, mitotic index, tumor rupture, Ki67 index, and adjuvant therapy were crucial independent prognostic factors for small intestinal GISTs. For patients with tumor size greater than 10 cm, mitotic index greater than 5, the occurrence of tumor rupture, high Ki67 positive index (>5%), closer follow-up is significant. Adjuvant treatment is necessary for surgical patients with intermediate and high-risk recurrence. Therefore, we suggest secondary prevention should be performed as soon as possible after surgery for patients with the above risk factors to obtain for obtaining a better prognosis. The relationship of tumor rupture and the Ki67 index with a poor prognosis in small intestinal GISTs should receive more attention. These parameters may improve not only the accurate risk assessments, but also lead to more individualized therapeutic and rigorous follow-up strategies in small intestinal GISTs.

## Acknowledgment

The authors acknowledge the support of the Liaoning province science and Technology Agency (no. 2014023034).

## Author contributions

**Conception and design:** Xin Chen, Hui Qu, and ZhaoHui Xu.

**Literature search and study selection:** Hui Qu and ZhaoHui Xu.

**Data collection:** Hui Qu, ZhaoHui Xu, George Kanani, ZeZhong Gong, and Yanying Ren.

**Analysis and interpretation:** Hui Qu, ZhaoHui Xu, Yanying Ren, Shuai Shao, and Ri Hyok Ju.

**Writing the article:** Hui Qu, ZhaoHui Xu, Fan Zhang, ZeZhong Gong, and Ri Hyok Ju.

**Critical revision of the article:** All authors.

**Final approval of the article:** All authors.

## References

[1] Kindblom LG Remotti HE Aldenborg F Meis-Kindblom JM. Gastrointestinal pacemaker cell tumor (GIPACT): gastrointestinal stromal tumors show phenotypic characteristics of the interstitial cells of Cajal. Am J Pathol 1998;152:1259–69. 9588894 PMC1858579

[2] Søreide K Sandvik OM Arne Søreide J Giljaca V Jureckova A Ramesh Bulusu V. Global epidemiology of gastrointestinal stromal tumours (GIST): a systematic review of population-based cohort studies. Cancer Epidemiol 2016;40:39–46. 26618334 10.1016/j.canep.2015.10.031

[3] Corless CL Barnett CM Heinrich MC. Gastrointestinal stromal tumours: origin and molecular oncology. Nat Rev Cancer 2011;11:865–78. 22089421 10.1038/nrc3143

[4] Pidhorecky I Cheney RT Kraybill WG Gibbs JF. Gastrointestinal stromal tumors: current diagnosis, biologic behavior, and management. Ann Surg Oncol 2000;7:705–12. 11034250 10.1007/s10434-000-0705-6

[5] Joensuu H Vehtari A Riihimäki J. Risk of recurrence of gastrointestinal stromal tumour after surgery: an analysis of pooled population-based cohorts. Lancet Oncol 2012;13:265–74. 22153892 10.1016/S1470-2045(11)70299-6

[6] Blanke CD Demetri GD von Mehren M. Long-term results from a randomized phase II trial of standard- versus higher-dose imatinib mesylate for patients with unresectable or metastatic gastrointestinal stromal tumors expressing KIT. J clin Oncol 2008;26:620–5. 18235121 10.1200/JCO.2007.13.4403

[7] Blanke CD Rankin C Demetri GD. Phase III randomized, intergroup trial assessing imatinib mesylate at two dose levels in patients with unresectable or metastatic gastrointestinal stromal tumors expressing the kit receptor tyrosine kinase: S0033. J Clin Oncol 2008;26:626–32. 18235122 10.1200/JCO.2007.13.4452

[8] Joensuu H Hohenberger P Corless CL. Gastrointestinal stromal tumour. Lancet 2013;382:973–83. 23623056 10.1016/S0140-6736(13)60106-3

[9] DeMatteo RP Lewis JJ Leung D Mudan SS Woodruff JM Brennan MF. Two hundred gastrointestinal stromal tumors: recurrence patterns and prognostic factors for survival. Ann Surg 2000;231:51–8. 10636102 10.1097/00000658-200001000-00008 PMC1420965

[10] Joensuu H. Risk stratification of patients diagnosed with gastrointestinal stromal tumor. Hum Pathol 2008;39:1411–9. 18774375 10.1016/j.humpath.2008.06.025

[11] Liu X Qiu H Zhang P. Ki-67 labeling index may be a promising indicator to identify “very high-risk” gastrointestinal stromal tumor: a multicenter retrospective study of 1022 patients. Hum Pathol 2018;74:17–24. 28962945 10.1016/j.humpath.2017.09.003

[12] Seven G Kochan K Caglar E Kiremitci S Koker IH Senturk H. Evaluation of Ki67 index in endoscopic ultrasound-guided fine needle aspiration samples for the assessment of malignancy risk in gastric gastrointestinal stromal tumors. Dig Dis 2021;39:407–14. 33017820 10.1159/000511994

[13] Belev B Brčić I Prejac J. Role of Ki-67 as a prognostic factor in gastrointestinal stromal tumors. World J Gastroenterol 2013;19:523–7. 23382631 10.3748/wjg.v19.i4.523 PMC3558576

[14] Casali PG Abecassis N Aro HT. Gastrointestinal stromal tumours: ESMO-EURACAN Clinical Practice Guidelines for diagnosis, treatment and follow-up. Ann Oncol 2018;29:iv68–78. 29846513 10.1093/annonc/mdy095

[15] Dematteo RP Gold JS Saran L. Tumor mitotic rate, size, and location independently predict recurrence after resection of primary gastrointestinal stromal tumor (GIST). Cancer 2008;112:608–15. 18076015 10.1002/cncr.23199

[16] Yang Z Wang F Liu S Guan W. Comparative clinical features and short-term outcomes of gastric and small intestinal gastrointestinal stromal tumours: a retrospective study. Sci Rep 2019;9:10033. 31296939 10.1038/s41598-019-46520-1 PMC6624285

[17] Patel DJ Lutfi W Eguia E. Adjuvant systemic therapy for small bowel gastrointestinal stromal tumor (GIST): is there a survival benefit after R0 resection? Surgery 2020;168:695–700. 32713755 10.1016/j.surg.2020.04.069

[18] von Mehren M Benjamin RS Bui MM. Soft tissue sarcoma version 2.2012: featured updates to the NCCN guidelines. J Natl Compr Cancer Netw 2012;10:951–60. 10.6004/jnccn.2012.0099 22878820

[19] Koo DH Ryu M-H Kim K-M. Asian consensus guidelines for the diagnosis and management of gastrointestinal stromal tumor. Cancer Res Treat 2016;48:1155–66. 27384163 10.4143/crt.2016.187 PMC5080813

[20] Grover S Ashley SW Raut CP. Small intestine gastrointestinal stromal tumors. Curr Opin Gastroenterol 2012;28:113–23. 22157511 10.1097/MOG.0b013e32834ec154

[21] Miettinen M Lasota J. Gastrointestinal stromal tumors: pathology and prognosis at different sites. Semin Diagn Pathol 2006;23:70–83. 17193820 10.1053/j.semdp.2006.09.001

[22] Joensuu H Rutkowski P Nishida T. KIT and PDGFRA mutations and the risk of GI stromal tumor recurrence. J Clin Oncol 2015;33:634–42. 25605837 10.1200/JCO.2014.57.4970

[23] Casali PG Zalcberg J Le Cesne A. Ten-year progression-free and overall survival in patients with unresectable or metastatic GI stromal tumors: long-term analysis of the European Organisation for Research and Treatment of Cancer, Italian Sarcoma Group, and Australasian Gastrointestinal Trials Group Intergroup Phase III randomized trial on imatinib at two dose levels. J Clin Oncol 2017;35:10.1200/JCO.2016.71.0228 28362562

[24] Tran T Davila JA El-Serag HB. The epidemiology of malignant gastrointestinal stromal tumors: an analysis of 1,458 cases from 1992 to 2000. Am J Gastroenterol 2005;100:162–8. 15654796 10.1111/j.1572-0241.2005.40709.x

[25] Mantese G. Gastrointestinal stromal tumor: epidemiology, diagnosis, and treatment. Curr Opin Gastroenterol 2019;35:555–9. 31577561 10.1097/MOG.0000000000000584

[26] Rizzo FM Palmirotta R Marzullo A. Parallelism of DOG1 expression with recurrence risk in gastrointestinal stromal tumors bearing KIT or PDGFRA mutations. BMC cancer 2016;16:87. 26867653 10.1186/s12885-016-2111-x PMC4750215

[27] Miettinen M Lasota J. Gastrointestinal stromal tumors: review on morphology, molecular pathology, prognosis, and differential diagnosis. Arch Pathol Lab Med 2006;130:1466–78. 17090188 10.5858/2006-130-1466-GSTROM

[28] Turkel Kucukmetin N Cicek B Saruc M. Ki67 as a prognostic factor for long-term outcome following surgery in gastrointestinal stromal tumors. Eur J Gastroenterol Hepatol 2015;27:1276–80. 26275084 10.1097/MEG.0000000000000454

[29] Seidal T Edvardsson H. Expression of c-kit (CD117) and Ki67 provides information about the possible cell of origin and clinical course of gastrointestinal stromal tumours. Histopathology 1999;34:416–24. 10231416 10.1046/j.1365-2559.1999.00643.x

[30] Jeong SY Park WW Kim YS. Prognostic significance of Ki-67 expression in patients undergoing surgical resection for gastrointestinal stromal tumor. Korean J Gastroenterol 2014;64:87–92. 25168050 10.4166/kjg.2014.64.2.87

[31] Fletcher CD Berman JJ Corless C. Diagnosis of gastrointestinal stromal tumors: a consensus approach. Hum Pathol 2002;33:459–65. 12094370 10.1053/hupa.2002.123545

[32] Rutkowski P Nowecki ZI Michej W. Risk criteria and prognostic factors for predicting recurrences after resection of primary gastrointestinal stromal tumor. Ann Surg Oncol 2007;14:2018–27. 17473953 10.1245/s10434-007-9377-9

[33] Gastrointestinal stromal tumors: ESMO Clinical Practice Guidelines for diagnosis, treatment and follow-up. Ann Oncol 2012;23 Suppl 7:vii49–55. 22997454 10.1093/annonc/mds252

[34] Güller U Tarantino I Cerny T Schmied BM Warschkow R. Population-based SEER trend analysis of overall and cancer-specific survival in 5138 patients with gastrointestinal stromal tumor. BMC Cancer 2015;15:557. 26223313 10.1186/s12885-015-1554-9 PMC4518595

[35] Bischof DA Kim Y Dodson R. Conditional disease-free survival after surgical resection of gastrointestinal stromal tumors: a multi-institutional analysis of 502 patients. JAMA Surg 2015;150:299–306. 25671681 10.1001/jamasurg.2014.2881 PMC4703090

[36] Nishida T Cho H Hirota S. Clinicopathological features and prognosis of primary GISTs with tumor rupture in the real world. Ann Surg Oncol 2018;25:1961–9. 29752602 10.1245/s10434-018-6505-7 PMC5976711

[37] Takahashi T Nakajima K Nishitani A. An enhanced risk-group stratification system for more practical prognostication of clinically malignant gastrointestinal stromal tumors. Int J Clin Oncol 2007;12:369–74. 17929119 10.1007/s10147-007-0705-7

[38] Rutkowski P Bylina E Wozniak A. Validation of the Joensuu risk criteria for primary resectable gastrointestinal stromal tumour – the impact of tumour rupture on patient outcomes. Eur J Surg Oncol 2011;37:890–6. 21737227 10.1016/j.ejso.2011.06.005

[39] Nishida T Hølmebakk T Raut CP Rutkowski P. Defining tumor rupture in gastrointestinal stromal tumor. Ann Surg Oncol 2019;26:1669–75. 30868512 10.1245/s10434-019-07297-9 PMC6510879

[40] Joensuu H Eriksson M Sundby Hall K. One vs three years of adjuvant imatinib for operable gastrointestinal stromal tumor: a randomized trial. JAMA 2012;307:1265–72. 22453568 10.1001/jama.2012.347

[41] Joensuu H Eriksson M Sundby Hall K. Adjuvant imatinib for high-risk GI stromal tumor: analysis of a randomized trial. J Clin Oncol 2016;34:244–50. 26527782 10.1200/JCO.2015.62.9170

[42] Liu X Qiu H Zhang P. Prognostic factors of primary gastrointestinal stromal tumors: a cohort study based on high-volume centers. Chin J Cancer Res 2018;30:61–71. 29545720 10.21147/j.issn.1000-9604.2018.01.07 PMC5842234

[43] Boikos SA Pappo AS Keith Killian J. Molecular subtypes of KIT/PDGFRA wild-type gastrointestinal stromal tumors: a report from the National Institutes of Health Gastrointestinal Stromal Tumor Clinic. JAMA Oncol 2016;2:922–8. 27011036 10.1001/jamaoncol.2016.0256 PMC5472100

[44] Szucs Z Thway K Fisher C. Molecular subtypes of gastrointestinal stromal tumors and their prognostic and therapeutic implications. Future Oncol 2017;13:93–107. 27600498 10.2217/fon-2016-0192

